# *Prom1* Function in Development, Intestinal Inflammation, and Intestinal Tumorigenesis

**DOI:** 10.3389/fonc.2014.00323

**Published:** 2014-11-14

**Authors:** Baktiar O. Karim, Ki-Jong Rhee, Guosheng Liu, Kyuson Yun, Steven R. Brant

**Affiliations:** ^1^Department of Molecular and Comparative Pathobiology, The Johns Hopkins University, Baltimore, MD, USA; ^2^Department of Biomedical Laboratory Science, Yonsei University, Gangwon-do, Wonju, South Korea; ^3^The Jackson Laboratory, Bar Harbor, ME, USA; ^4^Department of Medicine, The Johns Hopkins University, Baltimore, MD, USA

**Keywords:** prominin1, CD133, IBD, colitis-associated colon cancer, Apc Min mice

## Abstract

*Prom1/CD133* has been identified in colorectal, hepatocellular, and pancreatic cancer as a cancer stem cell marker and has been used as such to predict colon cancer recurrence in humans. Its potential molecular function as well as its role as a marker of intestinal regeneration is still not fully known. We evaluated the role of *Prom1* in intestinal regeneration in inflammatory bowel disease (IBD), determined the function of *Prom1*, and characterized the effect of a lack of *Prom1* on intestinal tumor formation in animal models. Our results suggest that *Apc* mutations lead to an increase in *Prom1* expressing cells in the intestinal crypt stem cell compartment and in early intestinal adenomas. Also, *Prom1* knockout mice are more susceptible to intestinal tumor formation. We conclude that *Prom1* likely plays a role in regulating intestinal homeostasis and that these results clearly illustrate the role of *Prom1* in intestinal regeneration. We further conclude that *Prom1* may provide a novel therapeutic target for patients with gastrointestinal conditions such as IBD, short bowel syndrome, and colorectal cancer.

## Introduction

The intestinal tract is lined by mucosa, which is primarily composed of villi or the intercrypt table and epithelial-lined crypts at the mucosal base (1–4). The mucosal epithelial cells arise from intestinal stem cells located in the crypt (5). The morphological and functional characterization of intestinal stem cells could shed light on their role in intestinal regeneration. The Clever laboratory found that PROM1 is a marker for stem cells and early progenitors in the mouse small intestine and that it meets all the criteria of putative intestinal stem cells (6). However, its role as a marker of intestinal regeneration is largely unknown.

PROM1 is a cell surface membrane protein and is a homolog to the CD133 protein in human (7). PROM1 was identified as both a hematopoietic and neuroepithelial stem cell marker (8–11). The function of Prom1 is unknown, but its specific localization suggests that PROM1 is involved in the organization of plasma membrane protrusions, and suggests that PROM1 might be important in maintaining an appropriate lipid composition within the plasma membrane (12). In addition, PROM1 has been identified in the apical plasma membrane of epithelial cells of pancreas (13), liver (canals of Hering, interlobular ducts) (14), kidney (15), prostate (16), and skin (17). In the small intestine in mouse, Prom1 marks rare stem cells, as well as transit-amplifying progenitor cells (6). Moreover, PROM1 is expressed in both rod and cone photoreceptors of the eye (18, 19). Prom1 also has been used as a cancer stem cell marker alone or with other markers such as CD44 (20). Singh and Dirks found that PROM1 expressing neural tumor cells are essential for tumorigenesis (21). Tang et al. found that PROM1 positive cell populations are more tumorigenic than PROM1 negative cells in the liver (22). In colon cancer, both PROM1 positive and PROM1 negative cells are capable of being tumorigenic (23). However, PROM1 expression in colon cancer in humans was found to be predictive of increased colon cancer recurrence (24, 25).

Wnt signaling regenerative pathways control intestinal differentiation (26, 27). Upon Wnt pathway activation, β-catenin stabilizes and translocates to the nucleus where it accumulates. It then, in cooperation with the transcription factor Tcf-4, modulates expression of a variety of Tcf-4 responsive target genes such as Paneth cell alpha defensins (28–30). However, whether *Prom1* expression is downstream of the Wnt and β-catenin/TCF transactivation activity is not clear.

Inflammation in the colon can play a significant role in colon cancer development (31). For example, the significantly increased risk of developing colon cancer in patients with long-standing inflammatory bowel disease (IBD) has been thought in large part to result from chronic colonic inflammation (31). The physiologic role of Prom1 in intestinal inflammation related to colon cancer development is not known. Characterization of Prom1 holds significant promise for eventual treatment of intestinal tumors and many gastrointestinal diseases such as Crohn’s Disease. We hypothesize that the Wnt signaling regenerative pathway results in dysregulation of Prom1 expression and subsequent changes in intestinal mucosal repair, and hence may be relevant to colon cancer development in IBD. To test this hypothesis, we investigated the role of Prom1 in intestinal regeneration and tumorigenesis. To gain insights into Prom1, we generated Prom1 knockout mice. Prom1 knockout mice were more susceptible to azoxymethane (AOM)/dextran sodium sulfate (DSS) induced intestinal inflammation, which promoted colorectal tumorigenesis. Also, loss of Prom1 gene contributed to higher tumorigenicity in Apc mutant mice.

## Materials and Methods

### Animal treatments

Wild type (*Wt*), *Prom1* knockout mice (*Prom1^-/-^*), and *Apc* mutant mice (*Apc^-/^*^+^) ranging from 1 day to 5 months old were used. *Wt* and 129-*Apc^tm1.^*^Δ^*^716^* mice were bred in our animal facility (27). *Prom1^-/-^* mice were provided by Dr. Kyuson Yun (un-published). To generate double mutant *Apc^-/^*^+^*Prom1^-/-^* mice, 129-*Apc^-/^*^+^ were crossed with 129-*Prom1*^−/−^ mice. All animals were maintained on a 129SvEv background (>10 generations). Pups were genotyped by PCR to determine *Apc* and *Prom1* gene status. The wild-type *Prom1* allele generated a 204-bp product, and the mutant allele formed a 329-bp product. Mice were fed an AIN-76A diet and water *ad libitum*, exposed to 12-h-light/12-h-dark cycles, and maintained under specific pathogen free conditions without pinworms, *Helicobacter spp*, or *Citrobacter rodentium*. We used age- and sex-matched littermate controls. All animal experiments were performed in accordance with protocols approved by the Animal Care and Use Committee of the Johns Hopkins Medical Institutions.

### Analysis of intestinal adenoma

At the age of 4 months, *Apc^-/^*^+^*Prom1^-/-^* and *Apc^-/^*^+^*Prom1*^+^*^/^*^+^ female mice were euthanized by cervical dislocation. The intestines were opened, stained with methylene blue (Figure 7A), and the number of adenomas was counted as previously described (27).

### Aberrant crypt foci

Aberrant crypt foci (ACF) were induced using AOM and DSS. Six-week-old female *Wt* and *Prom1^-/-^* mice (*N* = 5 per group) fed an AIN-76A diet and water *ad libitum* were treated with a single dose of AOM (2.5, 5, 7.5, or 10 mg/kg in saline, i.p.). Mice were assessed every 12 h for 5 days. Another group of *Wt* and *Prom1^-/-^* mice (*N* = 10 per group) were treated with a single dose of AOM (5 mg/kg in saline, i.p.). Seven days after injection with AOM, the mice were given normal drinking water or drinking water containing 2% DSS for a period of 7 days followed by normal drinking water for 3 weeks. ACF in surviving mice were evaluated as previously described with minor modifications (32). Intestinal lesions were scored for the presence of inflammation, mucosal damage, and proliferation. Grade 0, normal intestine; Grade 1, mild mixed infiltration of inflammatory cells; Grade 2, moderate infiltration of mixed inflammatory cells and edema within the mucosal layer and mild epithelial crowding; Grade 3, moderate to severe inflammation, edema, mucosal disruption, and crypt proliferation (epithelial crowding, deep crypts, and thicken mucosa); and Grade 4, severe transmural inflammation, edema, mucosal disruption, and severe proliferation (thickened mucosa, elongated and branched crypts, loss of goblet cells).

### Serum chemistry and blood examination

At the age of 6 weeks, blood was taken from both *Wt* and *Prom1^-/-^* mice. Both serum and blood were sent to Antech Diagnostics for analysis. Serum was analyzed for liver, kidney, and pancreas functions as well as muscle changes. The blood exam included total red blood cells (RBC), total white blood cells (WBC), and differential WBC counts. Thrombin, partial thrombin, hemoglobin, packed cell volume, mean corpuscular hemoglobulin (MCH), and mean corpuscular hemoglobulin concentration (MCHC) were also measured.

### Immunofluorescence and immunohistochemical staining

The adult *Wt, Prom1^−/−^*, and *Apc^-/^*^+^ mice were euthanized by cervical dislocation. The intestines were opened longitudinally, washed in PBS, and fixed in 10% buffered formalin. Then, the intestines were rolled and submitted for embedding. Five-micrometer-thick sections were prepared, and sections were deparaffinized in xylene and rehydrated through graded alcohols. Slides were transferred to a jar containing unmasking solution (Vector Laboratories, H-3300), boiled for 10 min, and left in the same solution at room temperature for 20 min. All slides were then incubated with 10% blocking serum (Vector Laboratories), in PBS for 30 min at room temperature. The slides were incubated with primary antibody (anti-Prom1, MAB4310, or with anti-Ki67, AB9260, Millipore) diluted 1:100 for 60 min at room temperature. After three washes with 0.1% Tween 20 in PBS, sections were incubated for 30 min with fluorescence (for Prom1) or non-fluorescence (for Ki67) biotinylated secondary antibody IgG (Vector laboratories) diluted 1:500 in blocking solution. Slides were washed three times in PBS for 3 min each, and rinsed three times with distilled water. Cover slips were mounted with crystal mount (Biomeda, M02). The number of positive cells within the crypts and adenomas was quantified in 10 fields at 400× magnification for Prom1 and at 200× magnification for Ki67. Photographs of histological sections were taken using a Nikon digital camera.

### Western blot analysis

Western blot analyses were performed on SDS-PAGE gels under denaturing conditions. Total proteins (30 μg/ml) from both *Wt* and *Prom1^-/-^* mice from intestinal crypt preparations were separated on 12 or 15% gels and electrotransferred onto nitrocellulose membranes (BioRad, 162-0115). Non-specific protein binding was blocked using 10% Blotto–0.1% Tween followed by incubation with anti-Prom1 primary antibody (Millipore, MAB4310) diluted in the blocking solution overnight at 4°C. After washing with PBS, membranes were incubated with horseradish peroxidase-conjugated anti-mouse secondary antibodies and developed using the Immobilon Western Kit (Millipore, WBKLS0100).

### Real-time PCR

Real-time PCR was performed to determine Prom1 expression on the crypt cells from both *Wt* and *Prom1^-/-^*. Total RNA of mouse intestinal tissues were extracted using the RNAeasy Kit (Invitrogen), and cDNA was synthesized using random hexamers. The Taqman primers for the *Prom1* and *K19* genes were from Applied Biosystems. We calculated relative gene expression by the ΔCT method.

### Data analysis

Data are presented as the mean and SEM or as a percentage with SD (GraphPad, PRISM software, San Diego, CA, USA). The Mann–Whitney test was used to compare body weights, and *P* values were determined using the Student two-tailed *t*-test unless otherwise indicated. Tumor data were analyzed using the Chi square (χ^2^) test and other differences using the Student’s *t*-test. Kaplan–Meier survival curves were generated, and difference in survival was determined using the log-rank test. Data were considered to be significantly relevant at *p* < 0.05 and are presented as mean ± SEM.

## Results

### Identification and characterization of Prom1 in normal intestinal crypt stem cell compartments and in early premalignant lesions in mice

**Figure 1:**
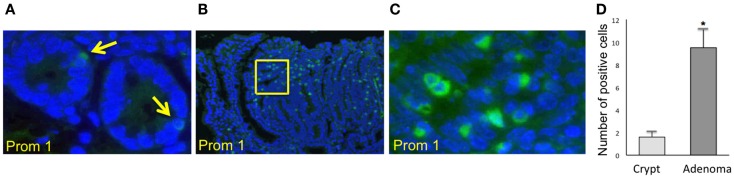
**Immunofluorescence staining using PROM1 in antibodies for the detection of expanded positive cells from crypt cells in Wt mice and within the adenomas in Apc^−/+^ mice**. **(A)** Normal crypt (magnification, ×600). **(B)** Adenoma (magnification, ×100). **(C)** Higher magnification (×600) of the image **(B)**. **(D)** Quantification analysis of the positive cells within the crypt and adenomas in the *Apc*^−/+^ mice. Bar = SEM. *p < 0.01 (*n* = 10).

**Figure 2:**
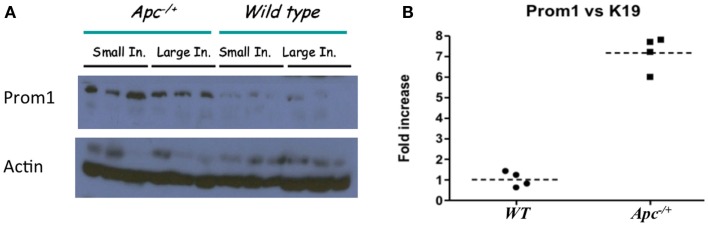
**Affect of Apc^−/+^ on Prom1 expression**. **(A)** Protein expression of PROM1 from small and large intestine in *Wt* mice and *Apc*^−/+^ mutant mice from three different mice. **(B)** mRNA expression of *Prom1* from large intestine of *Wt* and *Apc*^−/+^ mice (*n* = 4).

Immunofluorescence staining for the normal epithelial cell crypts in both the small and the large intestines revealed that anti-PROM1 antibody marked rare intestinal crypt cells (Figure 1A). In the *Apc^-/^*^+^ mice, that anti-PROM1 antibody showed intense staining in the majority of the epithelial cells within the adenomas (Figures 1B,C). The isotype matched control was negative (data not shown). Quantification showed an expansion of PROM1 positive cells in the adenomas, *P* < 0.001 (Figure 1D). Western blot analysis showed that mucosal cells from *Apc^-/^*^+^ mice have an enhanced expression of PROM1 in comparison to normal crypts from wild-type mice (Figure 2A). Real-time PCR was performed on the crypt cells using *K19* or *Gadph* (data not shown for *Gadph*) as controls. Results demonstrated significant increases (four- to six-fold) in *Prom1* expression in the crypt epithelial cells in the *Apc^-/^*^+^ mice compared to the crypts from wild-type mice (Figure 2B). These results indicate that *Prom1* expression and PROM1 positive cells are increased in the mucosa of *Apc^-/^*^+^ mice.

### Role of *Prom1* during normal growth and development in a knockout animal model system

**Figure 3:**
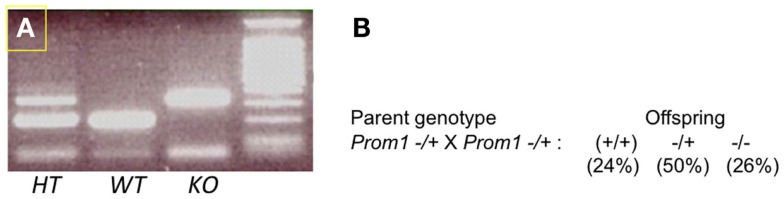
**Genotypes of offspring obtained by cross-mating of *Prom1^−/+^* and *Prom1^−/+^* double heterozygous mice**. **(A)** PCR of Prom1 heterozygous, wild type, and homozygous genomic DNA to detect mutant allele. **(B)** Mendelian ratios, single gene (*Prom1*) knockout.

**Figure 4:**
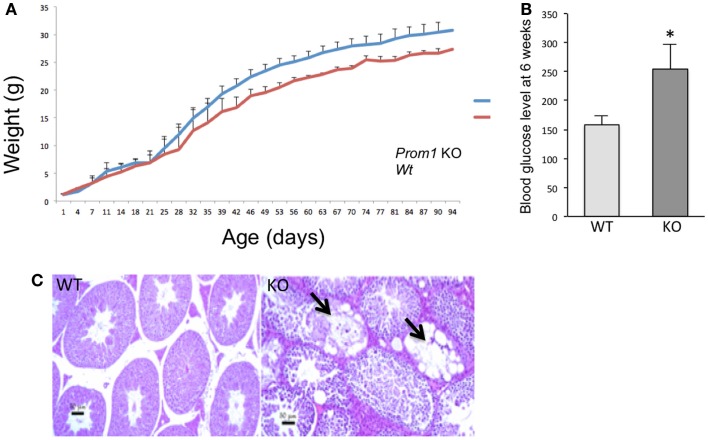
**Phenotype of Prom1 knockout mice**. **(A)** Body weight change from day 1 to day 94. **(B)** Blood glucose level at 6 weeks of age in *Wt* and *Prom1*^−/−^ mouse (*n* = 10). **(C)** Histopathological examination of the testes at 4 months of age showing empty seminiferous tubules in the *Prom1*^−/−^ mouse (arrow), H&E ×100.

To determine the role of *Prom1* in intestinal crypt, we analyzed *Prom1^-/-^* mice. Null mice of both sexes were phenotypically normal, and crossing heterozygous male and female mice resulted in *Wt*, *Het*, and null offspring at the expected Mendelian ratios (Figure 3). Body weight analysis revealed mature obesity in *Prom1^-/-^* mice compared to *Wt* mice (Figure 4A). Complete histopathological analysis revealed that there is a moderate degree of germinal arrest in the testes of *Prom1^-/-^* mice compared to wild-type mice. There was multifocal seminiferous tubule atrophy and degeneration and arrest of spermatogenesis within the testes in the *Prom1^-/-^* mice at 4 months of age (Figure 4C). Serum chemistry revealed a significant increase in fasting blood glucose level in *Prom1^-/-^* mice. The mean blood glucose level was 254 mg/dl in the *Prom1^-/-^* mice compared to 158 mg/dl in the wild-type mice (Figure 4B). The knockout did not affect hematology. There were no changes in blood scores in the *Prom1^-/-^* mice compared to wild-type mice (data not shown).

### Influence of *Prom1^-/-^* genotype on intestinal mucosal healing and proliferation during intestinal injury in mice

**Figure 5:**
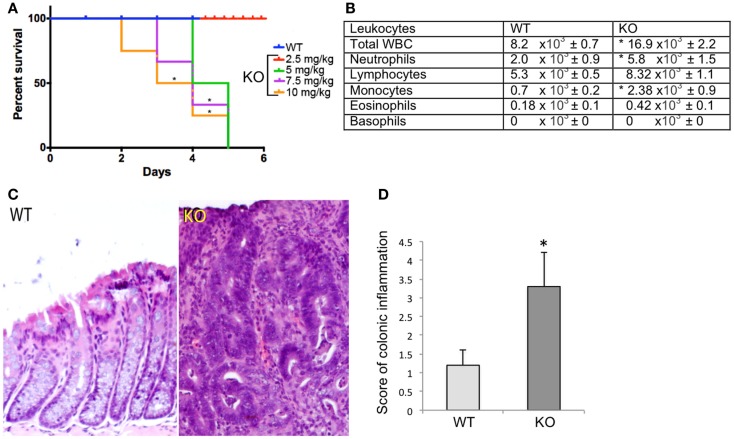
**Significant differences in survival and inflammation in the Wt and *Prom1^−/−^* treated group**. **(A)** Survival curve of *Wt* and *Prom1*^−/−^ mice after AOM administration that was assessed every 12 h for 5 days. **(B)** Peripheral leukocyte counts of *Wt* and *Prom1*^−/−^ mice after AOM/DSS administration. **(C)** H&E of large intestine in *Wt* and *Prom1*^−/−^mice after AOM/DSS administration. Notice: inflammation, dysplastic crypts, and abnormal proliferation in knockout mice. **(D)** Scores of inflammation in the inflamed colon. (*p < 0.05 compared with control, *n* = 6 animals/group).

**Figure 6:**
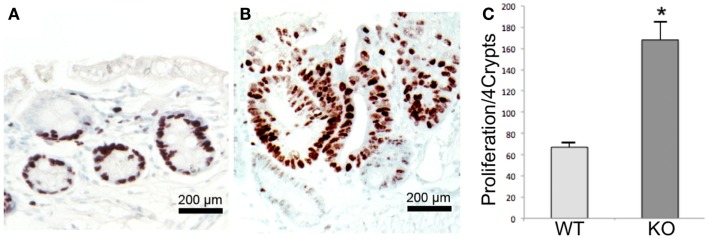
**Proliferation assay in Wt and Prom1^−/−^ mice after AOM/DSS administration**. Immunohistochemical staining of Ki67 within the crypts in *Wt* **(A)** and *Prom1*^−/−^ **(B)** mice. **(C)** Quantification analysis of Ki 67 staining. Bar = SEM. *p < 0.01 (*n* = 10).

To determine whether Prom1 has a role in maintaining intestinal epithelium homeostasis and injury response, we exposed control and *Prom1^-/-^* to AOM and DSS. *Prom1^-/-^* mice were more sensitive to AOM than *Wt* mice. After injection with a single dose of AOM (5 mg/kg), 50% of the mice died. All the *Prom1^-/-^* mice died when given a single injection of AOM at 7.5 or 10 mg/kg (Figure 5A). Remarkably, an injection of AOM followed by oral exposure to DSS resulted in increased intestinal inflammation in *Prom1^-/-^* mice, relative to *Wt* mice (Figures 5C,D). Inflammatory infiltrate consists predominantly of macrophages, neutrophils, and lesser numbers of lymphocytes and rare plasma cells. In addition to increase in inflammation, *Prom1^-/-^* mice showed severe dysplastic crypts and abnormal crypt proliferation. Hyperplastic crypts in the knockout mice showed markedly reduced number of goblet cells, elongated nuclei with prominent nucleoli, and abnormal crypt lumen. The wild-type mice exhibited mild crypt hyperplasia with minimal dysplasia. Peripheral complete blood counts revealed significant leukocytosis, neutrophilia, and monocytosis in *Prom1* knockout mice compared with the wild-type mice (Figure 5B). The proliferation index of hyperplastic crypts measured by nuclear expression of Ki67 showed a significant positive correlation to the lack of *Prom1*, *p* < 0.01 (Figure 6).

### Effect of Prom1-/- on intestinal tumor growth

**Figure 7:**
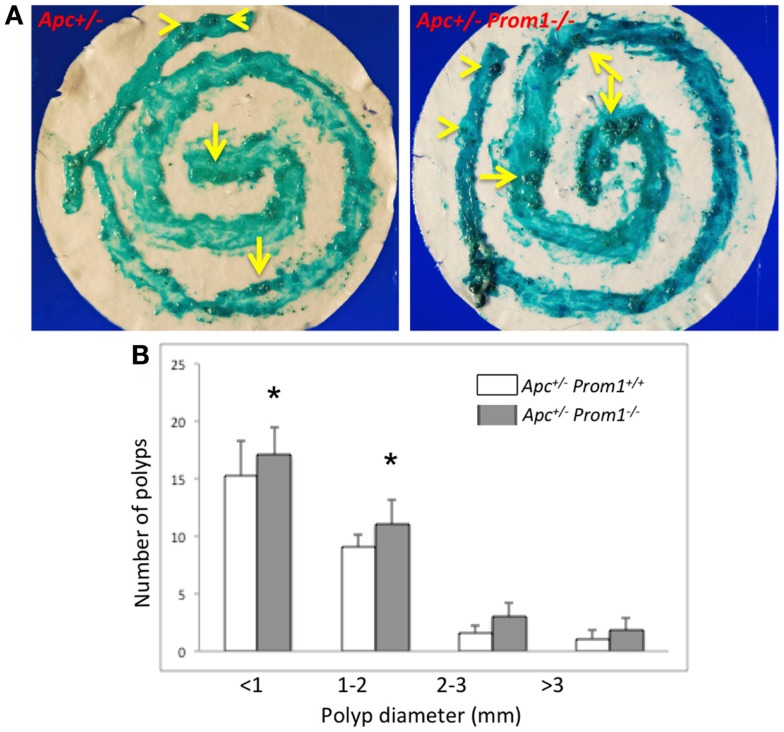
**(A)** Methylene blue staining of the entire intestine is shown. Notice various sized of adenomas within the large (arrow head) and small intestine (arrows). **(B)** Distribution of adenomas by size in *Apc^-/^*^+^ *prom1*^+^*^/^*^+^ (white bar) and in *Apc^-/^*^+^ *Prom1^-/-^* mice (filled bar). The percentage of control adenomas is indicated with SEM. The number of adenomas was analyzed by the Chi-square test and Student’s *t*-test (*p < 0.05 versus control, *n* = 15 animals/group).

To determine whether loss of Prom1 promotes intestinal tumorigenesis, we crossed *Prom1^-/-^* mice to *Apc^-/^*^+^ mice. All sizes of adenomas increased in the double mutant mice (*Apc^-/^*^+^*Prom1^-/-^*) (Figure 7A), especially small adenomas (<2 mm in diameter) (Figure 7B). Double mutant mice exhibited the highest number of adenomas (mean, 33) compared with *Apc*^−/+^ mice (mean, 27) along their entire small and large intestines at ~120 days of age. Average adenoma count was 23 in the small intestine *and* 10 in the large intestine of double mutant mice (*Apc^-/^*^+^*Prom1^-/-^*) compared to 17 and 10 in single mutant mice (*Apc^-/^*^+^*Prom*^+^*^/^*^+^), respectively. The number of adenomas increased by 18% in *Apc^-/^*^+^*Prom1^-/-^* mice compared to those with *Apc^-/^*^+^*Prom*^+^*^/^*^+^. This escalation was highly significant as mediated by a *p* value of <0.05 using a two-tailed Student’s *t*-test.

## Discussion

Despite medical advances, IBD remains a significant and increasing health care burden worldwide, and the exact cause remains unknown (33). However, intestinal regeneration plays an important role in the healing of the intestinal mucosa (34). We hypothesized that PROM1 is important for intestinal homeostasis. The results reported here demonstrate that Prom1 plays an important role in intestinal healing. Knockout of *Prom1* results in significantly greater intestinal inflammation, abnormal crypt proliferation, and dysplasia upon AOM/DSS administration. Inflammation in general promotes intestinal tumorigenesis (35). The use of PROM1 to mark the intestinal stem cells and colorectal cancer is controversial (36). Researchers found that both PROM1^+^ cells and PROM1^–^ cells are capable of tumorigenicity and display similar differentiation capabilities. For example, Liqin Zhu found that Prom1 marks stem cells in the adult small intestine that are susceptible to transformation into tumors (37). In contrast, Meng et al. discovered that PROM1 alone could not be used as a stem cell marker because PROM1^+^ cells and PROM1^−^ cells displayed similar abilities of colony formation, self-renewal, proliferation, and differentiation (38). Our results show that the loss of *Prom1* combined with *Apc* gene heterozygosity significantly increases tumorigenesis. Together, these results suggest that *Prom1* functions as a protective factor against early phase, inflammation-mediated tumorigenesis.

Several investigators found that PROM1 is a target for Wnt signaling regenerative pathways in many cell lines including malignant melanoma and glioblastoma cells (39, 40). In the absence of Wnt, Apc forms a complex in the cytoplasm that results in β-catenin phosphorylation by glycogen synthase kinase-3 (GSK-3). This results in proteolytic degradation of β-catenin. Loss of Apc function and GSK-3 phosphorylation results in stabilized β-catenin (41, 42). This in turn allows β-catenin to translocate to the nucleus and accumulate, where, in cooperation with the transcription factor Tcf-4, it modulates expression of a variety of Tcf-4 responsive target genes such as Paneth cell α-defensins (28–30). However, it was not clear whether *Prom1* behaves as a positive or negative regulator in mice having *Apc* mutations. Our results are consistent with previous studies showing that PROM1 is expressed predominantly on the crypt stem cell compartment. We now show that PROM1 can also be detected in early premalignant lesions in *Apc^-/^*^+^ mice under Wnt signaling regenerative pathways and that *Prom1^-/-^* deletion stimulates adenomas in *Apc^-/^*^+^ mice, which is a part of Wnt signaling regenerative pathways. These data indicate that *Prom1^-/-^* is a potential target for chemoprevention treatment. The anticancer activity of PROM1 is unknown, and that is beyond the scope of this paper. Taken together, our results suggest that *Prom1^-/-^* induces inflammation and increases proliferative potential in the intestinal crypts enhanced intestinal tumor genesis.

Knockout of *Prom1* did not affect early development in mice; *Prom1^-/-^* disruption did not cause any lethal embryonic defects or interfere with development or fertility, although we did observe compromised spermatogenesis in some Prom1^−/−^ males. Histopathological analysis concluded that *Prom1* is essential for maintenance of healthy testicular tissue. Also, *Prom1^-/-^* mice were significantly obese and had increased in fasting blood glucose as compared with control to *Wt* mice. Therefore, there appears to be a link between *Prom1* and pancreatic islet cell function. Epidemiological associations of both diabetes and obesity with colon cancer risk have been well-established (43). It will be of great interest to determine if decreased *Prom1* presence or activity has a role in the relationships between diabetes or obesity and risk for colon cancer in humans. Review manuscript suggests that there is a link between colon cancer progression and obese individual due to mitochondrial dysfunction (44, 45).

To our knowledge, this is the first study to provide an in-depth evaluation of the role and function of *Prom1*. Given the strong influence of *Prom1* presence and control of progression of inflammation and development of dysplastic crypts, as well as limiting tumorigenesis in the presence of *Apc* loss, one may hypothesize that PROM1 may have a role in inflammation-mediated colonic dysplasia such as that which occurs not infrequently in long-standing IBD of the colon in humans. It will be of great interest to determine if PROM1 presence or activity is reduced in human IBD and particularly in IBD-associated dysplasia and colon cancer. Our findings may also imply that accommodation of the *Prom1* pathway by small molecules might be a useful chemopreventive strategy in long-standing IBD. Our results also raise additional questions to be addressed in future studies regarding Prom1 function: if overexpression of *Prom1* in proliferative intestinal crypts is mediated by Wnt signaling regenerative pathways and if *Prom1* expression acts as a negative regulator in intestinal tumorigenesis, what role does Prom1 play in Wnt responses? Furthermore, does expression of Prom1 contribute to the maintenance of “cancer stemness”?

## Conflict of Interest Statement

The authors declare that the research was conducted in the absence of any commercial or financial relationships that could be construed as a potential conflict of interest.
